# Pediatric Cancer Communication on Twitter: Natural Language Processing and Qualitative Content Analysis

**DOI:** 10.2196/52061

**Published:** 2024-05-07

**Authors:** Nancy Lau, Xin Zhao, Alison O'Daffer, Hannah Weissman, Krysta Barton

**Affiliations:** 1 Center for Child Health, Behavior and Development Seattle Children’s Research Institute Seattle, WA United States; 2 Department of Psychiatry and Behavioral Sciences University of Washington School of Medicine Seattle, WA United States; 3 Department of Psychiatry University of California, San Diego San Diego, CA United States; 4 Center for Empathy and Technology Sanford Institute for Empathy and Compassion University of California, San Diego San Diego, CA United States; 5 Department of Psychology Vanderbilt University Nashville, TN United States; 6 Biostatistics Epidemiology and Analytics for Research (BEAR) Core Seattle Children’s Research Institute Seattle, WA United States

**Keywords:** cancer, COVID-19, Twitter, communication, child health, caregivers, social media, tweet, tweets, sentiment, oncology, cancers, pediatric, pediatrics, child, children’ youth, experience, experiences, attitude, attitudes, opinion, opinions, perception, perceptions, perspective, perspectives

## Abstract

**Background:**

During the COVID-19 pandemic, Twitter (recently rebranded as “X”) was the most widely used social media platform with over 2 million cancer-related tweets. The increasing use of social media among patients and family members, providers, and organizations has allowed for novel methods of studying cancer communication.

**Objective:**

This study aimed to examine pediatric cancer–related tweets to capture the experiences of patients and survivors of cancer, their caregivers, medical providers, and other stakeholders. We assessed the public sentiment and content of tweets related to pediatric cancer over a time period representative of the COVID-19 pandemic.

**Methods:**

All English-language tweets related to pediatric cancer posted from December 11, 2019, to May 7, 2022, globally, were obtained using the Twitter application programming interface. Sentiment analyses were computed based on Bing, AFINN, and NRC lexicons. We conducted a supplemental nonlexicon-based sentiment analysis with ChatGPT (version 3.0) to validate our findings with a random subset of 150 tweets. We conducted a qualitative content analysis to manually code the content of a random subset of 800 tweets.

**Results:**

A total of 161,135 unique tweets related to pediatric cancer were identified. Sentiment analyses showed that there were more positive words than negative words. Via the Bing lexicon, the most common positive words were support, love, amazing, heaven, and happy, and the most common negative words were grief, risk, hard, abuse, and miss. Via the NRC lexicon, most tweets were categorized under sentiment types of positive, trust, and joy. Overall positive sentiment was consistent across lexicons and confirmed with supplemental ChatGPT (version 3.0) analysis. Percent agreement between raters for qualitative coding was 91%, and the top 10 codes were awareness, personal experiences, research, caregiver experiences, patient experiences, policy and the law, treatment, end of life, pharmaceuticals and drugs, and survivorship. Qualitative content analysis showed that Twitter users commonly used the social media platform to promote public awareness of pediatric cancer and to share personal experiences with pediatric cancer from the perspective of patients or survivors and their caregivers. Twitter was frequently used for health knowledge dissemination of research findings and federal policies that support treatment and affordable medical care.

**Conclusions:**

Twitter may serve as an effective means for researchers to examine pediatric cancer communication and public sentiment around the globe. Despite the public mental health crisis during the COVID-19 pandemic, overall sentiments of pediatric cancer–related tweets were positive. Content of pediatric cancer tweets focused on health and treatment information, social support, and raising awareness of pediatric cancer.

## Introduction

Social media platforms are widely used to exchange information and share resources. One such platform is Twitter (recently rebranded as “X”), a microblogging site with approximately 400 million global users. Social media platforms such as Twitter have been used by patients with health conditions, their caregivers, and other family members to connect with individuals in similar situations and learn from patients, researchers, and organizations worldwide. Patients with cancer, survivors of cancer, and their family members commonly use Twitter as a resource for treatment information and social support. Twitter users consist of a variety of cancer stakeholders including cancer centers, pharmaceutical companies, nonprofit organizations, medical providers, patients, and patients’ family and friends [1]. Individuals and organizations also use Twitter and other social media platforms to increase awareness and reach of cancer-related messages [2]. The increasing use of social media among patients, providers, and organizations has allowed for novel ways of studying cancer communication [3].

The global COVID-19 pandemic led to major changes in lifestyle, social distancing, and isolation that uniquely affected patients with cancer, caregivers, and other stakeholders. They were negatively impacted by overly taxed health care infrastructure and medical systems, restricted access to medical care, and a mental health crisis. Research on cancer during the pandemic spanned a range of topics including the global impact of COVID-19 on cancer care management [4,5]. Cancer survivors’ stressors during the pandemic included anxiety about in-person appointments, fear of cancer recurrence, medical care delays, uncertainty about future medical care, untreated symptoms, and mental health concerns [6]. Caregivers of patients with pediatric cancer experienced changes to their child’s medical care, financial disruptions, and emotional stress related to COVID-19 [7]. The COVID-19 pandemic was also associated with an increased risk of depression and loneliness in people living with cancer [8].

Twitter has been the most frequently used social media platform for public health surveillance since 2006, and there were over 2 million cancer-related tweets during the pandemic [9,10]. Previous studies have examined changes in public sentiment and the increasing use of Twitter during the pandemic [11,12]. For example, an analysis of Twitter showed that patients with cancer expressed significant negative sentiment during the COVID-19 pandemic [9]. Recent studies have examined the content of cancer-related tweets for different types of cancer diagnoses including lung, breast, and prostate cancer [13-15]. We only identified 2 studies thus far that have examined the pediatric cancer experience on Twitter. The first was a cross-sectional study examining the use of Twitter to discuss childhood cancer during Childhood Cancer Awareness Month [16]. The second used Twitter data to conduct a lexicon-based sentiment analysis of patients with pediatric cancer using the hashtag #ChildhoodCancer and found generally positive sentiment scores [17].

Lexicon-based sentiment analytic approaches determine positive and negative sentiments based on individual words [18]. Recent behavioral health studies have used lexicon-based approaches to analyze short text messages on social media platforms such as Twitter [19-23]. In addition to determining the positive or negative sentiment of words, the “NRC” lexicon assigns a sentiment type using the following additional emotion categories: anger, anticipation, disgust, fear, joy, sadness, surprise, and trust [18]. Such analyses provide population-level insights into patterns of health information sharing and support-seeking on social media, and can inform the dissemination of time-critical information and resources during challenging times such as the COVID-19 pandemic. Additionally, the launch of Open AI’s chatbot, ChatGPT, provides a novel tool for nonlexicon-based sentiment analysis via text-based chat inquiries. Emerging research suggests that ChatGPT demonstrates superior performance in sentiment analysis of free-text responses [24].

In this study, we examined cancer-related tweets for pediatric cancer globally to capture the experiences of patients and survivors of cancer, their caregivers, medical providers, and other stakeholders. The objectives of our analysis using a Twitter data set over a time period representative of the impact of the COVID-19 pandemic were two-fold: (1) to quantitatively analyze the public sentiment of tweets related to pediatric cancer via lexicon-based and nonlexicon-based sentiment analytic approaches; and (2) to qualitatively examine the topics relevant to cancer diagnosis, treatment, and survivorship covered with hashtags commonly associated with pediatric cancer via a directed content analysis.

## Methods

### Ethical Considerations

This study did not require institutional review board approval because we used publicly available social media data that do not involve human subjects and do not fall within the scope of Human Subjects Research. Seattle Children’s Hospital’s Institutional Review Board uses the US Department of Health and Human Services (DHHS) definition of Human Subjects Research. Human Subjects Research under DHHS regulations is defined as the investigator obtaining information through intervention or interaction with living individuals; or obtaining, using, studying, analyzing, or generating identifiable private information. Our research is Nonhuman Subjects research according to the Seattle Children’s HRP-101 Human Research Protection Program Plan, P. 3, which uses the DHHS definition of Human Subjects Research [25,26]. The publicly available social media data reported in this paper have been anonymized and contains no IDs, user names, or nonparaphrased tweets.

### Data Collection

In this study, we examined pediatric cancer–related communication on Twitter encompassing a representative timeframe ranging from before to after the COVID-19 pandemic. We obtained a total of 182,628 publicly available global tweets from December 11, 2019, to May 7, 2022, using the Twitter application programming interface. An example of the query and timeline information using “#teenagecancer” is available in Multimedia Appendix 1. For this study, we restricted our collection to English-only tweets. We identified a list of hashtags commonly associated with pediatric cancer: #childhoodcancer, #childhoodcancerawareness, #childhoodcancerday, #internationalchildhoodcancerday, #kidsgetcancertoo, #pediatriccancer, #pediatriconcology, #teenagecancer. These 8 keywords were selected because they were representative of hashtags frequently used for pediatric cancer. The prepandemic period was designated as December 2019 to February 2020. The pandemic time period was designated as March 2020 to June 2020. Lockdown and mandatory stay-at-home orders were issued in 42 US states and territories across the United States between March and May 2020 during the height of the pandemic [27]. The postpandemic time period was designated as July 2020 to May 2022 after mandatory stay-at-home orders were lifted in all states across the United States. Removing duplicates resulted in a total of 161,135 tweets from 40,289 unique users. All unique tweets were used for lexicon-based sentiment analysis. Among the 161,135 tweets from 40,289 unique accounts, we then randomly sampled a subset of 800 tweets and analyzed them using a directed content approach. Of the subset of tweets, 300 were randomly sampled and proportionately stratified by pandemic period (prepandemic, during the pandemic, and postpandemic).

### Sentiment Analysis

#### Overview

“Sentiment analysis” or “opinion mining” is a natural language processing technique used to analyze and extract insights from text data, enabling the identification and understanding of the sentiment, emotions, and subjective opinions expressed within the text, which can be valuable for various applications such as market research, customer feedback analysis, and social media monitoring. We used lexicon-based approaches to conduct analyses using the full data set of tweets. Nonlexicon-based approaches can be used to evaluate whether the results may align with lexicon-based analysis. Thus, we used ChatGPT to conduct supplemental analyses on a randomly selected subsample of tweets.

#### Lexicon-Based Approaches

All data preprocessing, cleaning, and analyses were performed in R (version 4.2.2; R Foundation for Statistical Computing). We used “saotd” for preprocessing and initial analyses [28]. Nonlanguage elements such as symbols, weblinks, punctuation, emojis, and stop words, such as “the” and “of,” were removed. Sentiment scores were first computed based on the Bing lexicon, and we presented the most common positive and negative words within the data set. Additional analyses were conducted using the “syuzhet” package. We computed sentiments based on “Bing,” “AFINN,” and “NRC” lexicons. The “Bing” lexicon was developed by Liu [29] and categorizes 6788 English words into positive and negative categories. The “NRC” lexicon includes 6468 English words and classifies words as positive or negative sentiments and includes emotional categories of anger, anticipation, disgust, fear, joy, sadness, surprise, and trust [30]. The “AFINN” lexicon includes 2476 English words that were labeled with a value between –5 (negative sentiment) and +5 (positive sentiment) [31]. We used the “get_sentiment” function in the “syuzhet” package to calculate sentiment scores. Final sentiment scores were generated for each of the lexicons. All positive sentiment scores for “Bing,” “AFINN,” and “NRC” lexicons were recoded to 1 and all negative sentiment scores were recoded to –1. Weekly average scores were calculated to reflect the average sentiment of tweets in a given week. We used the “plot_ly” function in the “plotly” package to visualize changes in weekly sentiment over time.

#### Supplemental Nonlexicon-Based Approach

ChatGPT (version 3.0) is a next-generation artificial intelligence–based chatbot optimized for using natural language processing to generate responses to user input [32]. We asked ChatGPT (version 3.0) to analyze the overall sentiments of a subset of 150 randomly sampled tweets, with 50 tweets each from our pre-, during-, and postpandemic data sets. We entered, “Can you provide the overall sentiments of the following tweets?” into the query box. ChatGPT responded: “I'd be happy to help you analyze the overall sentiments of the tweets you've provided” along with its conclusions on sentiment. We analyzed the sentiment of 50 tweets per data set which was below the maximum size data set allowed for the free version of ChatGPT.

### Qualitative Coding

We explored the background literature related to cancer and other health conditions to identify a codebook based on directed content analysis for our project [13,33-36]. We identified Sutton et al [13] for lung cancer messages as the codebook that was most relevant and related to our sample of pediatric cancer tweets. We conducted a directed content analysis [37] using codes and coding definitions from Sutton et al’s codebook. Further, 2 of the authors (NL and AO) coded tweets in sets of 10 to iteratively refine and adapt the Sutton et al codebook and definitions to correspond to pediatric cancer-related tweets. We expanded the preliminary codebook to include the emergence of 7 new coding categories that did not exist in the original codebook. We also added a not enough information coding category for tweets that were ambiguous and could not be coded. Furthermore, 2 of the authors (NL and AO) met weekly to discuss and address codebook discrepancies, and further refine the codebook. The entire authorship team met to discuss codebook development and refinement until it was stable and finalized. The same 2 coders (NL and AO) used the final codebook of content of tweets (adapted from Sutton et al [13]; Textbox 1) to independently double-code all 800 of the randomly sampled tweets.

**Final version of qualitative codebook of content of tweets.**

**Research**

- Text that describes research on cancer at any point in the continuum, including study results, study in progress, conference presentations, journal publications, research gaps, news publications describing recent findings, and researcher profiles. Any media source, for example, internet blogs, WebMD, or consumer-focused articles apply.

**Awareness**

- Text that promotes awareness of cancer (eg, fundraising and prevalence), discusses potential symptoms and signs of cancer, activism, philanthropy, inequities, books, or memoirs about the cancer experience, or makes general references to cancer.

**Policy and the law**

- Text about insurance, benefits, legal issues, public policy, and government funding. Code policy and the law only if the tweet does not contain additional content that would lead you to double-code as awareness or another code.

**Pharmaceuticals and drugs**

- Text that mentions a generic or brand name drug or a pharmaceutical firm.

**Prevention and risk information**

- Text that describes cancer risk, behaviors that increase risk (eg, smoking and environmental causes), and behaviors that reduce risk or prevent cancer (eg, healthy diet and smoking cessation).

**Early detection**

- Text that describes screening tests (eg, low-dose computed tomography), warning signs, early symptoms, and family history.

**Diagnosis**

- Text that contains information about a diagnosis, such as tests (eg, imaging, tests, and biopsy) and results (eg, malignant or benign).

**Treatment**

- Text that describes attempts to medically remove or alter cancer or cancer symptoms (eg, chemotherapy and surgery), discusses treatment of symptoms, references individuals receiving treatment (eg, “fighting cancer”), or information about potential treatments.

**Survivorship**

- Text that describes life after cancer treatment, including remission, and long-term effects of treatment.

**Mental health**

- Text that describes the impact of the cancer journey on mental health, mental health treatment or resources, and mental health support.

**End of life**

- Text that discusses cancer-related deaths and legacy. Supportive messages, remembrances, and condolences regarding a patient who died. Parents tweeting about their own children who died of cancer.

**Personal experiences**

- Text that mentions a personal experience with cancer, including messages about the self and others who have experienced cancer or are worried about cancer. Includes publicized memoirs. If unclear identity of the tweet author (eg, patient, caregiver, and provider), only code personal experiences.

**Patient experiences**

- Text from individuals with pediatric cancer diagnosis regarding self-experiences. Double-code with personal experiences.

**Caregiver experiences**

- Text from caregiver of pediatric cancer regarding personal experiences. Double-code with personal experiences.

**Health status**

- Text that describes current health status, illness progression, and related effects (eg, worries, concerns, and hope).

**Social support**

- Supportive messages to a patient or caregiver in their illness journey. Encouraging messages from survivors of cancer to other survivors.

**Provider experiences**

- Supportive messages and appreciation for specific providers who care for patients with cancer or providers in general. Clinicians discussing their experiences providing care. Double-code with personal experiences.

**Not enough information**

- Not enough information in the tweet to code content.

Interrater reliability was calculated as the percent agreement between raters before consensus meetings. Consensus conversations occurred weekly and we referenced the codebook to resolve any discrepancies. The qualitative data were analyzed using DeDoose (Sociocultural Research Consultants, LLC) software for code frequency counts and code co-occurrences. Data visualization of codes was represented by a word cloud generated in DeDoose.

### Research Team

Authors’ backgrounds included health services research (NL, XZ, AO, HW, and KB), digital health research (NL, XZ, and AO), analytics (XZ), implementation science (NL), clinical psychology (NL, XZ, and AO), pediatric psychology (NL and XZ), bioethics (KB), qualitative research (NL, XZ, and KB), and psychosocial oncology research (NL, AO, HW, and KB).

## Results

### Sentiment Analyses

**Figure 1:**
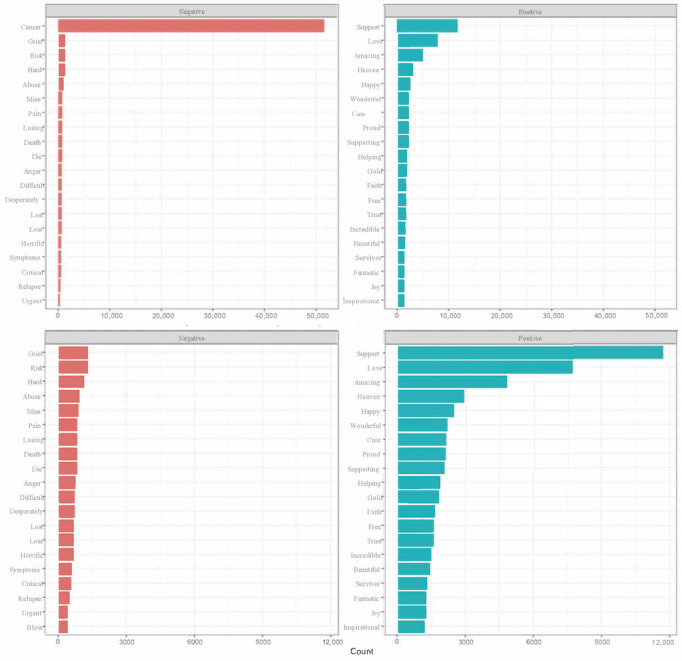
Most common positive and negative words using the Bing lexicon.

**Figure 2:**
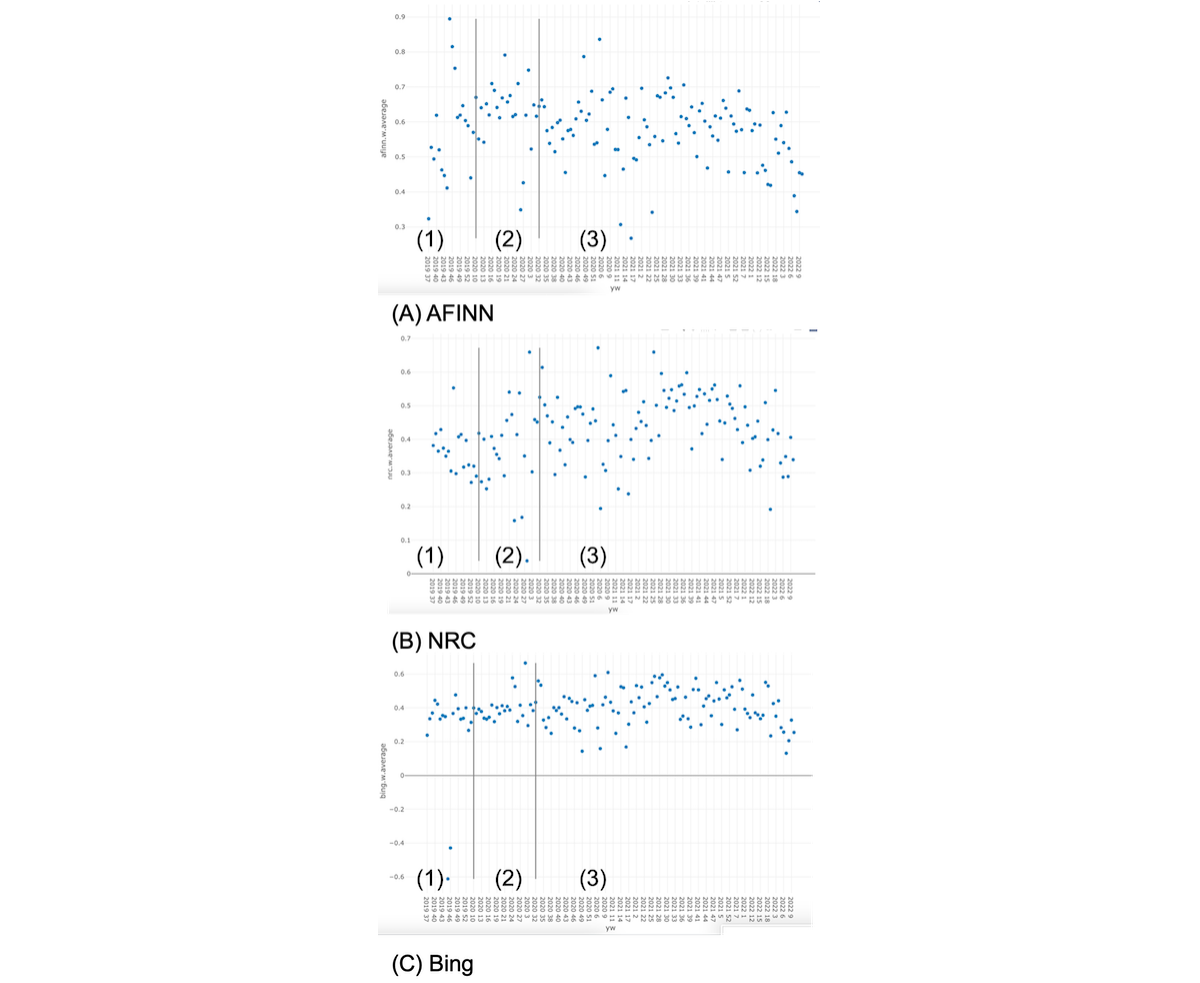
Weekly sentiment using different lexicons from December 11, 2019, to May 7, 2022, a time period representative of the impact of the COVID-19 pandemic. (1) Pre–COVID-19 pandemic, (2) during–COVID-19 pandemic, and (3) post–COVID-19 pandemic.

Cancer was the most commonly mentioned word, as it was included in all hashtags that were used to collect the tweets. Excluding “cancer,” “Bing” lexicon-based sentiment analyses revealed that there were more positive words than negative words in the extracted tweets (Figure 1). The “Bing” lexicon was based on the largest lexicon among our 3 lexicons and was able to analyze the largest number of tweets. The 5 most commonly observed positive words in the “Bing” lexicon and our data set were “support,” “love,” “amazing,” “heaven,” and “happy.” The 5 most commonly observed negative words in the “Bing” lexicon and our data set were “grief,” “risk,” “hard,” “abuse,” and “miss.” Analyses from the “NRC” lexicon showed that most tweets were categorized under the sentiment types of “positive” (N=138,752), “trust” (N=101,036), and “anticipation” (N=100,635). Figure 2A-C displays weekly sentiment scores over a time period representative of the impact of the COVID-19 pandemic for the “Bing,” “AFINN,” and “NRC” lexicons. The sentiment was overall positive. These findings were consistent across lexicons. Based on responses from ChatGPT (version 3.0), the randomly selected subsamples from the pre-, during-, and postpandemic periods demonstrated overall positive sentiment (Textbox 2). Although ChatGPT (version 3.0) analysis was exploratory, findings were consistent with our lexicon-based analyses.

**Overall sentiments provided by ChatGPT in supplemental analysis.**

**Pre–COVID-19 pandemic**

- The overall sentiments in the provided texts are predominantly positive or neutral. The texts largely revolve around messages of support, encouragement, and raising awareness for childhood cancer, which are inherently positive causes. There is an emphasis on helping and supporting children with cancer and celebrating their milestones. Overall, the texts convey sentiments of empathy and goodwill toward individuals affected by childhood cancer, making the overall sentiment positive and empathetic.

**During–COVID-19 pandemic**

- Overall, the sentiment in most of these tweets is positive or neutral, as they primarily involve raising awareness, requesting support, or expressing gratitude for contributions to childhood cancer causes.

**Post–COVID-19 pandemic**

- Overall, this collection of tweets has a predominantly positive and neutral sentiment with some mixed and concerned sentiments. The positivity in these tweets largely stems from support for childhood cancer-related causes and achievements in the field.

### Qualitative Coding

Percent agreement between coders was high (91%) before consensus meetings. Consensus meetings resolved all coding discrepancies. The top 5 codes were awareness (N=449), personal experiences (N=166), research (N=60), caregiver experiences (N=54), patient experiences (N=53), policy and the law (N=52), treatment (N=21), end of life (N=21), pharmaceuticals and drugs (N=17), and survivorship (N=15). Data visualization of code applications in a word cloud using DeDoose software is presented in Multimedia Appendix 2.

Twitter users predominantly used the social media platform to promote public awareness of pediatric cancer. In addition, Twitter users frequently use the social media platform to share personal experiences with cancer. Many accounts were from the firsthand perspectives of caregivers of patients with pediatric cancer in active treatment, bereaved parents, and from patients or survivors of cancer themselves. Twitter was frequently used for health knowledge dissemination of research findings (topics included cancer prevention and risk information, early detection, diagnosis, treatment, and survivorship). Twitter was also frequently used to call attention to and lobby for government programs and federal policies that support pediatric cancer treatment and affordable medical care. Example tweets for the top 10 codes are presented in Textbox 3.

**Figure 3:**
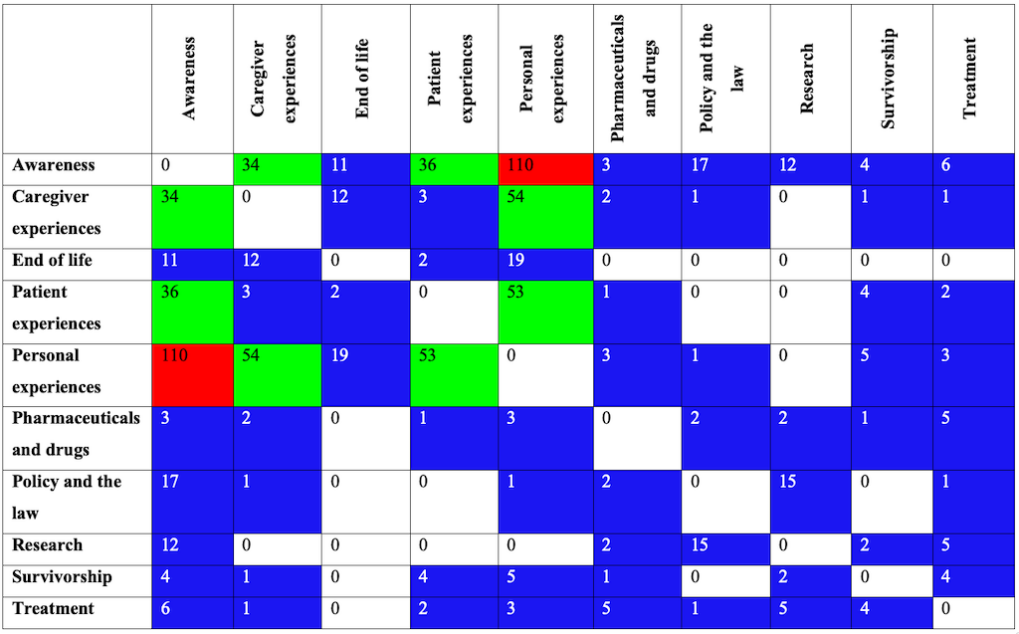
Code co-occurrence chart of top 10 coding categories generated by DeDoose.

DeDoose software displays the frequency of co-occurring codes using a heat map. Most frequently co-occurring codes are in red, moderately frequent co-occurring codes are in green, and less frequently co-occurring codes are in blue. The code co-occurrence chart of the top 10 codes is presented in Figure 3. Overwhelmingly, awareness and personal experiences were the most frequently cocoded (110 times). Moderately frequent co-occurring codes were caregiver experiences and personal experiences (cocoded 54 times), personal experiences and patient experiences (cocoded 53 times), awareness and patient experiences (cocoded 36 times), and awareness and caregiver experiences (cocoded 34 times).

**Example of paraphrased and deidentified tweets for Top 10 coding categories.**

**Awareness**

- GOLD is the symbolic color for #ChildhoodCancerAwareness. Wearing GOLD to show your support for our children! From head-to-toe, we want to see how gold you can be for Spirit Day! Tag us in your photos
- We are grateful for the impact on the #pediatriccancer world! Every donation makes a difference.

**Personal experiences**

- Please help get [this] story out there. #CancerSucks #ChildhoodCancerAwareness. she needs our help!!! RT and donate if you can. Thank you! *Awareness
- FAMily Update» [Child’s name] is back in the hospital. The medical team has ordered a 24 hour [inpatient stay]. #FAM #FightingAllMonsters #ChildhoodCancer *Caregiver Experiences; Health Status

**Research**

- Brain and spinal cord tumors are the 2nd most common cancers in children. In honor of #ChildhoodCancerAwareness month, here’s a look at recent #Umich discoveries to help treat brain cancer in kids. *Awareness; Treatment
- With more than 10,000 experts worldwide and nearly 100 active clinical trials across the spectrum of childhood cancers, COG is committed to ending #childhoodcancer as we know it. #ChildhoodCancerAwareness #ChildhoodCancerAwarenessMonth *Treatment

**Caregiver experiences**

- I’m involved with many amazing groups for bereaved parents (like myself) and many other #ChildhoodCancer groups. I’d love to see you join us. *End of Life; Personal Experiences
- I am blessed to be the [caregiver] of one of the toughest kids in the world. Love you. #InternationalChildhoodCancerDay #teensvscancer *Personal Experiences

**Patient experiences**

- [Child’s name] has [medical event] which landed him in the ER. Shout out your loudest prayers and coolest thoughts for [child’s name] so he can return home #FAM #FightingAllMonsters #ChildhoodCancer *Personal Experiences; Social Support
- Agree friends get nervous of saying wrong thing so tend to say nothing I was lucky had a couple of mates there throughout. *Personal Experiences; Social Support
- [Child’s name] finishes his radiotherapy tomorrow!! He put a smile on everyone's face with his [resilience/playfulness]! *Personal Experiences; Health Status

**Policy and the law**

- Please do not allow the Creating Hope Reauthorization Act S.4010 to die on YOUR watch! It has produced 28 drugs for rare pediatric diseases, My [child] received 1. *Pharmaceuticals & Drugs; Personal Experiences; Caregiver Experiences
- Please join Congressman Peter Welch VT-0 as a cosponsor of HR 6556 Gabriella Miller Kids First Research Act 2.0. No taxpayer funds required to help #ChildhoodCancer & rare diseases #GMKF2
- We need non-toxic therapies developed for #ChildhoodCancer which is not the same as adults. In the last 10 years, kid’s cancers received only 4% of the budgeted govt research. Will you help? *Awareness

**End of life**

- This time of year can be especially difficult for those who are grieving, esp. for parents who have lost a child. This is my [personal experience]. Here is some advice to help us get through. Please RT. #ChildhoodCancer #Grief #Grieving *Mental Health; Personal Experiences; Caregiver Experiences
- Nothing will ever be so awful as [child’s death]. I am very grateful to the NHS and @TeenageCancer for their efforts but they just couldn’t save him. *Personal Experiences; Caregiver Experiences
- Missing [child’s name] today. Please lend some support to this petition to fund research into childhood cancers #ChildhoodCancer #BrainTumourCharity *Awareness; Personal Experiences

**Treatment**

- A novel #CARTcelltherapy has shown promising early results in #children with #neuroblastoma, a rare form of ChildhoodCancer. #CancerImmunotherapy *Pharmaceuticals & Drugs
- Clear guidance for stem cell transplant patients, those who have had abdominal radiotherapy, and those who have had total body irradiation as part of transplant #coronavirus #COVID19 #childhoodcancer

**Pharmaceuticals and drugs**

- A NFCR partner working to advance new therapies for #childhoodcancer, @OncoHeroesBio, recently announced that the @US_FDA has granted Orphan Drug Designation and Rare Pediatric Disease Designation to one of its drugs. #Together4ACure
- Check out the article: “Leaving no child behind in the fight against cancer” A very good explanation on the current issues we face in #ChildhoodCancer drug development, as well as recommendations to solve the current issues! @SIOPEurope
- #ACCELERATEcure (virtual) Annual Conference 2021 - REGISTRATION open next week! Looking forward to welcoming you to discover latest developments worldwide in #ChildhoodCancer drug development! Join us *Research

**Survivorship**

- The list of potential side effects of #ChildhoodCancer treatments includes future fertility problems, visual loss, dental complications. With the right testing these side effects can be guarded against #ChildhoodCancerAwarenessMonth #Pharmacogenomics *Awareness; Treatment
- Substantial progress has been made against the most common types of pediatric cancers and overall survival rates are up, but more hard work remains so more children with cancer not only survive but thrive. #GoldTogether #ChildhoodCancer *Treatment

## Discussion

### Principal Results

The purpose of this study was to examine the communication content of pediatric cancer–related tweets and the public sentiment of pediatric cancer tweets. The sentiment of tweets on pediatric cancer was overall positive, revealing the supportive, hopeful, and inspirational messages relayed by patients, caregivers, and other relevant stakeholders. Our findings were consistent with the only other study to examine the public sentiment of pediatric cancer–related tweets [17]. Despite previous research showing predominantly negative sentiments globally during the COVID-19 pandemic [38], our study describes a positive sentiment of pediatric cancer–related tweets during the COVID-19 pandemic. We found that pediatric cancer–related tweets predominantly focused on raising awareness about pediatric cancer and disseminating health knowledge. We found that both patients with pediatric cancer or survivors and their caregivers frequently used Twitter to provide updates on their health status, for social support, and to share messages of hope.

We only identified 2 studies thus far that have examined the pediatric cancer experience on Twitter. The current study expanded on the growing body of literature on social media use in patients with cancer, and early research on pediatric cancer-related use of Twitter. Previous research studies have discussed the importance of including caregiver experiences in addition to those of the patient for individual and family-based well-being and adaptive coping [39-41]. Our findings were consistent with previous studies that have shown that cancer-related tweets center on health communication and social support [36]. Similar to previous studies, there was a diverse array of Twitter users, representing perspectives from patients, family members, oncology providers, and health care, pharmaceutical, nonprofit, and other organizations [2,36,42,43]. Additional studies may use combined sentiment analysis and qualitative approaches to better understand pediatric cancer communication and support resources on Twitter and other popular social media platforms. The current and future studies can help inform the development of novel patient- and caregiver-based social media interventions to improve health knowledge, change health behaviors, and improve health outcomes.

### Limitations

Our study has several limitations. First, our analysis included only tweets in the English language which limits generalizability to populations that do not speak English as a primary language. Second, we analyzed tweets that contained prespecified keywords (ie, hashtags) and may have missed other pediatric cancer–related tweets during the specified study period. Third, we only examined social media use on Twitter which may differ from usage on other popular social media platforms. Fourth, we were unable to identify the account type (organization vs individual, patient or caregiver vs researcher) and extract sociodemographic information of users; this information may have further informed our research findings and the conclusions drawn. Fifth, our qualitative content analysis of Twitter only included a random sample of tweets from the full data set which may not be representative of all pediatric cancer–related tweets during the specified timeframe of our analysis. Sixth, our lexicon-based approaches have inherent limitations. Despite using multiple sentiment lexicons in our analyses, such approaches analyze sentiment based on individual words. We did not expand contractions and apply stemming in our analyses as they were not available in “saotd,” the statistical package we used for data preprocessing. The lack of expanding contractions and applying stemming may have reduced the number of analyzable words and tweets. Twitter users commonly use contractions, abbreviations, slang, and sarcasm. Thus, we conducted supplemental analyses using ChatGPT (version 3.0), a next-generation artificial intelligence optimized for natural language processing, to validate our findings. Although exploratory, these findings were consistent with lexicon-based approaches. Research should further investigate the use of other recent innovative nonlexicon-based approaches that analyze entire sentences, such as embedding-based approaches or transformer-based approaches to analyze tweets related to pediatric cancer. Seventh, our data were global tweets but our specified “pre-,” “during-,” and “post-” pandemic time periods were based on United States lockdowns and timelines. We acknowledge that pandemic timelines differ within the United States and certainly globally. Nonetheless, we think it is important to include all tweets regardless of geographic location for representativeness of experiences due to the pandemic being a global crisis.

### Conclusions

Acute, ongoing, and evolving pediatric medical traumatic stress impacts the child in the context of their family, which emphasizes the importance of incorporating the perspectives and experiences of caregivers and other family members [44,45]. Social media use by patients with pediatric cancer, their families, and their medical providers has been well-described [46]. Uses and benefits include opportunities for social support, building collaborative networks, dissemination of health-related information, and treatment recommendations [46]. Researchers have increasingly turned to sentiment and content analyses of Twitter to capture real-time experiences of patients with a range of health conditions and other relevant stakeholders. Such research has included analyses of tweets about various cancer diagnoses.

Twitter, as a popular social media platform, may serve as an effective means for researchers to examine pediatric cancer communication and public sentiment around the world. This study used both quantitative and qualitative methods to examine the pediatric cancer experience on Twitter. Despite the global mental health crisis during the COVID-19 pandemic, we found overall positive sentiment of pediatric cancer–related tweets over a time period representative of the COVID-19 pandemic. The content of pediatric cancer tweets was posted by a range of users and centered on the delivery of health and treatment information, seeking and providing social support, and the amplification of awareness of pediatric cancer. Twitter may serve as a powerful platform for rapid communication with survivors of pediatric cancer and their caregivers, and facilitate the widespread dissemination of patient- and caregiver-targeted behavioral health interventions to improve well-being and quality of life. This would be well-matched to pediatric cancer survivors’ and their caregivers’ current preferences in social media use.

## Appendix

Multimedia Appendix 1 An example of the query and timeline information using the hashtag #teenagecancer.

Multimedia Appendix 2 Data visualization of code applications in a word cloud using DeDoose software.

## Abbreviations

DHHS: Department of Health and Human Services

## Data Availability

The publicly available data underlying this paper were accessed from Twitter. The derived data generated in this research will be shared on reasonable request to the corresponding author.
