# Supplementary figures and images for: Pediatric Cancer Communication on Twitter: Natural Language Processing and Qualitative Content Analysis
Source: JMIR Cancer. 2024 May 7;10:e52061. doi: 10.2196/52061 (PMC11109854; doi:10.2196/52061)

**Multimedia Appendix 1.** Query and timeline information of “”.


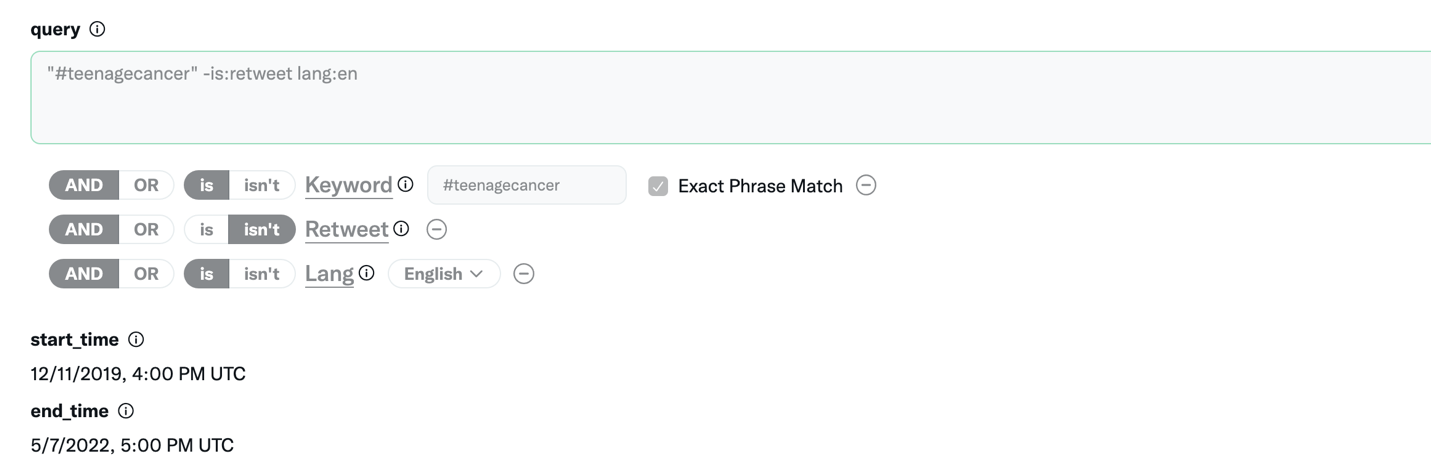

Supplement: Multimedia Appendix 1 [file cancer_v10i1e52061_app1.docx]

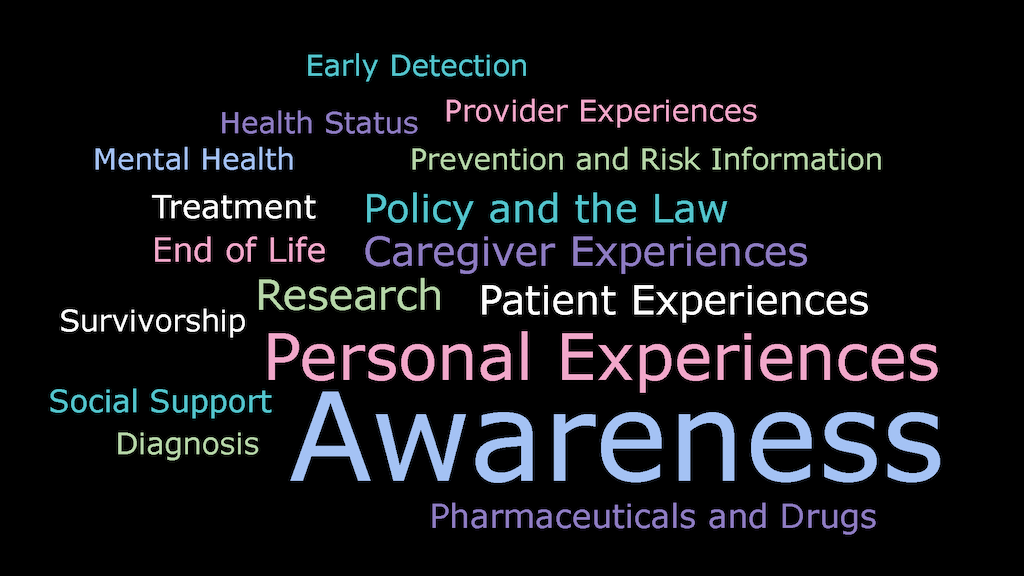

Supplement: Multimedia Appendix 2 [file cancer_v10i1e52061_app2.png]
